# Prominent Bone Loss Mediated by RANKL and IL-17 Produced by CD4+ T Cells in TallyHo/JngJ Mice

**DOI:** 10.1371/journal.pone.0018168

**Published:** 2011-03-25

**Authors:** Hee Yeon Won, Jin-Ah Lee, Zong Sik Park, Jin Sook Song, Hee Yun Kim, Su-Min Jang, Sung-Eun Yoo, Youmi Rhee, Eun Sook Hwang, Myung Ae Bae

**Affiliations:** 1 Division of Life and Pharmaceutical Sciences, College of Pharmacy, Center for Cell Signaling & Drug Discovery Research, Ewha Womans University, Seoul, Korea; 2 Korea Bio-Organic Science Division, Korea Research Institute of Chemical Technology, Daejeon, Korea; 3 School of Medicine, Yonsei University, Seoul, Korea; Ulm University, Germany

## Abstract

Increasing evidence that decreased bone density and increased rates of bone fracture are associated with abnormal metabolic states such as hyperglycemia and insulin resistance indicates that diabetes is a risk factor for osteoporosis. In this study, we observed that TallyHo/JngJ (TH) mice, a polygenic model of type II diabetes, spontaneously developed bone deformities with osteoporotic features. Female and male TH mice significantly gained more body weight than control C57BL/6 mice upon aging. Interestingly, bone density was considerably decreased in male TH mice, which displayed hyperglycemia. The osteoblast-specific bone forming markers osteocalcin and osteoprotegerin were decreased in TH mice, whereas osteoclast-driven bone resorption markers such as IL-6 and RANKL were significantly elevated in the bone marrow and blood of TH mice. In addition, RANKL expression was prominently increased in CD4+ T cells of TH mice upon T cell receptor stimulation, which was in accordance with enhanced IL-17 production. IL-17 production in CD4+ T cells was directly promoted by treatment with leptin while IFN-γ production was not. Moreover, blockade of IFN-γ further increased RANKL expression and IL-17 production in TH-CD4+ T cells. In addition, the osteoporotic phenotype of TH mice was improved by treatment with alendronate. These results strongly indicate that increased leptin in TH mice may act in conjunction with IL-6 to preferentially stimulate IL-17 production in CD4+ T cells and induce RANKL-mediated osteoclastogenesis. Accordingly, we propose that TH mice could constitute a beneficial model for osteoporosis.

## Introduction

Bone tissue continuously undergoes remodeling through mechanical coupling of osteoclastic bone resorption and osteoblastic bone formation. Bone homeostasis is controlled by a variety of soluble factors such as growth factors, hormones, and cytokines. For example, insulin-like growth factor (IGF), transforming growth factor (TGF) and parathyroid hormone (PTH) stimulate the production of bone collagen and matrix proteins in osteoblasts and increase osteoclast apoptosis, thus resulting in increased bone formation [1]–[6]. On the other hand, inflammatory cytokines, including IL-1, IL-6, and TNF-α, enhance osteoclastic bone resorption, further inducing bone destruction [7]–[10]. Osteoclast activity is directly stimulated by osteoblasts through the interaction of receptor activator of NF-κB (RANK) with RANK ligand (RANKL) [11], whereas osteoprotegerin (OPG), a decoy RANKL receptor produced by osteoblasts, inhibits osteoclast differentiation and activity [12], [13]. RANKL is expressed not only in osteoblasts but also in activated CD4+ T cells [14]–[16] and is increased by IL-1β or TNF-α in combination with IL-17 [17]. Other cytokines produced by T cells such as IL-4, IL-13, IFN-γ, and TGF-β induce OPG expression and interfere with the RANKL-RANK signaling pathway, thus suppressing osteoclastogenesis [18], [19].

Bone functions as an endocrine organ by producing bone hormone osteocalcin (OCN) [20]. Osteoblast-specific OCN is a hormone that increases insulin production and sensitivity, promoting glucose uptake and energy metabolism [21]. OCN deficiency reduces β-cell proliferation and insulin secretion, mediating insulin resistance [22]. Moreover, OCN functions *in vitro* on pancreatic β-islet cells to stimulate insulin production and secretion and on muscle to increase insulin-mediated glucose uptake [20]. Moreover, OCN modulates the expression of adiponectin and leptin in adipocytes, suggesting that it may control an intimate link between bone and fat [23]. Recent findings that OCN expression is associated positively with insulin sensitivity [24]–[26], as well as the observation that decreased bone density and increased fracture risk are closely associated with diabetes [27]–[30], strongly support the notion that bone homeostasis is affected by insulin insufficiency or resistance under diabetic conditions. Consistently, hyperglycemic and hyperleptinemic conditions are identified as osteoporotic risk factors. However, the molecular links between these risk factors and osteoporosis remain largely uncharacterized.

In this study, we observed that TH mice, a polygenic diabetes model, displayed severe osteoporotic phenotypes with male predominance and intended to uncover the molecular links between inflammatory factors and osteoporosis in TH mice. Whereas bone mineral density (BMD) and OCN were significantly decreased in TH mice, RANKL, IL-6, and IL-17 expression and osteoclast activity were considerably elevated. In addition, we observed that RANKL expression was increased in CD4+ T cells of TH mice and that blockade of IFN-γ and leptin further increased IL-17 production in CD4+ T cells. These results suggest that TH mice displaying bone loss as well as increased levels of IL-17 and RANKL in T cells due to hyperleptinemia may be an effective animal model for osteoporosis secondary to diabetes in males.

## Materials and Methods

### Ethics Statement

All animal studies were conducted in accordance with IACUC guidelines and were approved by the IACUC committee at Ewha Womans University (ELAGC-05-1007 and IACUC 2010-13-1).

### Animals

C57BL/6 (B6) and TallyHo/JngJ (TH) mice were purchased from Jackson Laboratory (Bar Harbor, ME, USA) and housed under specific pathogen-free conditions.

### Isolation and activation of CD4+ T cells

Single cell suspensions were obtained from the lymph nodes (LN) of B6 and TH mice and incubated with magnetic beads coated with anti-mouse CD4 Ab (Miltenyi Biotech, Auburn, CA, USA). CD4+ T cells were isolated according to the manufacturer's instructions and stimulated with anti-CD3 (1 µg/ml, BD Pharmingen, San Jose, CA, USA) and anti-CD28 (1 µg/ml, BD Pharmingen) Abs in the presence of recombinant human IL-2 (10 U/ml, BD Pharmingen) for 24 h or 48 h.

### Assessment of bone microarchitecture and measurement of BMD and BMC

B6 and TH mice were analyzed by high-resolution micro-computed tomography (micro-CT, Explore Locus Scanner, GE Healthcare, Ontario, Canada) with an 80 kV energy source, 450 µA current, 400 ms exposure time, and 46 µm pixel size. Approximately 400 projections were acquired over a rotation range of 180 degrees, with a rotation step of 1 degree. The femur was scanned at pixel size of 0.046 mm. The three-dimensional bone images were reconstituted using a micro-CT scanner (eXplore Ultra, GE Healthcare) and micro view image processing software (MicroView v2.2, GE Healthcare).

### *In vitro* differentiation of bone marrow cells and cell staining

Bone marrow-derived mononuclear cells (BMMCs) were isolated from the femurs and tibias of B6 and TH mice and cultured in DMEM supplemented with 10% fetal bovine serum (FBS). After removal of floating cells, BMMCs were cultured under osteogenic differentiation conditions for 8 days. Cells were fixed and then stained with either alkaline phosphatase (ALP) or tartrate-resistant acid phosphatase (TRAP) using a staining kit according to the manufacturer's instructions (Sigma-Aldrich, St. Louis, MO, USA).

### Reverse transcription and quantitative real-time PCR analysis

Total RNA was isolated using TRIzol reagent (Gibco-BRL, Invitrogen), followed by reverse transcription and real-time PCR reactions using Superscript II (Invitrogen) and SYBR Green pre-mix buffer and with an ABI-Prism 7700 sequence detector (Perkin-Elmer Applied Biosystems, Foster City, CA, USA). Primers were as follows: IL-6, 5′-agttgccttcttgggactga-3′, 5′- tccacgatttcccagagaac-3′; RANKL, 5′-ctcttggtaccacgatcgag-3′, 5′-aagccccaaagtacgtcgca-3′; OPG, 5′-atgccgagagtgtagagaggat-3′, 5′-aaacagcccagtggaccattcct-3′; OCN, 5′-gcagcttggtgcacacctag-3′, 5′-ggagctgctgtgacatccat-3′; IFN-γ, 5′-agcaacagcaaggcgaaaa-3′, 5′-ctggacctgtgggttgttga-3′; IL-17, 5′-gctccagaaggccctcaga-3′, 5′-agctttccctccgcattga-3′; aromatase, 5′-aaatgctgaaccccatgcag-3′, 5′-aatcaggagaaggaggcccat-3′; and β-actin, 5′-agagggaaatcgtgcgtgac-3′, 5′-caatagtgatgacctggccgt-3′. Relative expression levels were calculated after normalization to the level of β-actin.

### ELISA/EIA

Cytokines such as IL-17, IFN-γ, IL-6, and RANKL were determined using ELISA kits (R&D systems, Minneapolis, MN, USA). High-sensitivity ELISA/EIA kits were used to determine the serum levels of leptin (Millipore. Billerica, MA, USA), OCN (Biomedical Technologies, Inc., Stoughton, MA, USA), and OPG (R&D Systems). Serum IL-6, IFN-γ, and TGF-β were determined using an ELISA Ready-SET-Go kit (eBioscience, Inc., San Diego, CA, USA) and serum levels of testosterone and estradiol were determined using EIA kits from Cayman chemical (Charlotte, NC, USA).

### Flow cytometry analysis

Single cell suspensions were collected from tissues and suspended in FACS buffer (2% FBS in PBS). Cells were incubated with fluorescence-conjugated antibodies against mouse CD4, CD8, Ly6C, and RANKL (BD Pharmingen) followed by flow cytometry analysis using FACS Calibur (BD Biosciences, San Jose, CA, USA). For intracellular cytokine staining, cells were pretreated with monensin (4 µM) for 2 h before harvesting. Cells were fixed with 4% paraformaldehyde solution, rinsed with permeabilization buffer (0.1% saponin, 1% FBS in PBS), and then incubated with PE-conjugated anti-IFN-γ Ab (BD Pharmingen). Cell populations were analyzed using CellQuest software (BD Biosciences).

### Statistical analysis

The results are expressed as the mean ± SEM. Statistical significance was determined by one-way analysis of variance (ANOVA) or two-tailed unpaired Student's t-test. A P value less than 0.05 was considered to be statistically significant for all experiments.

## Results

### Bone density and bone mineral content (BMC) are substantially decreased in TH mice

**Figure 1:**
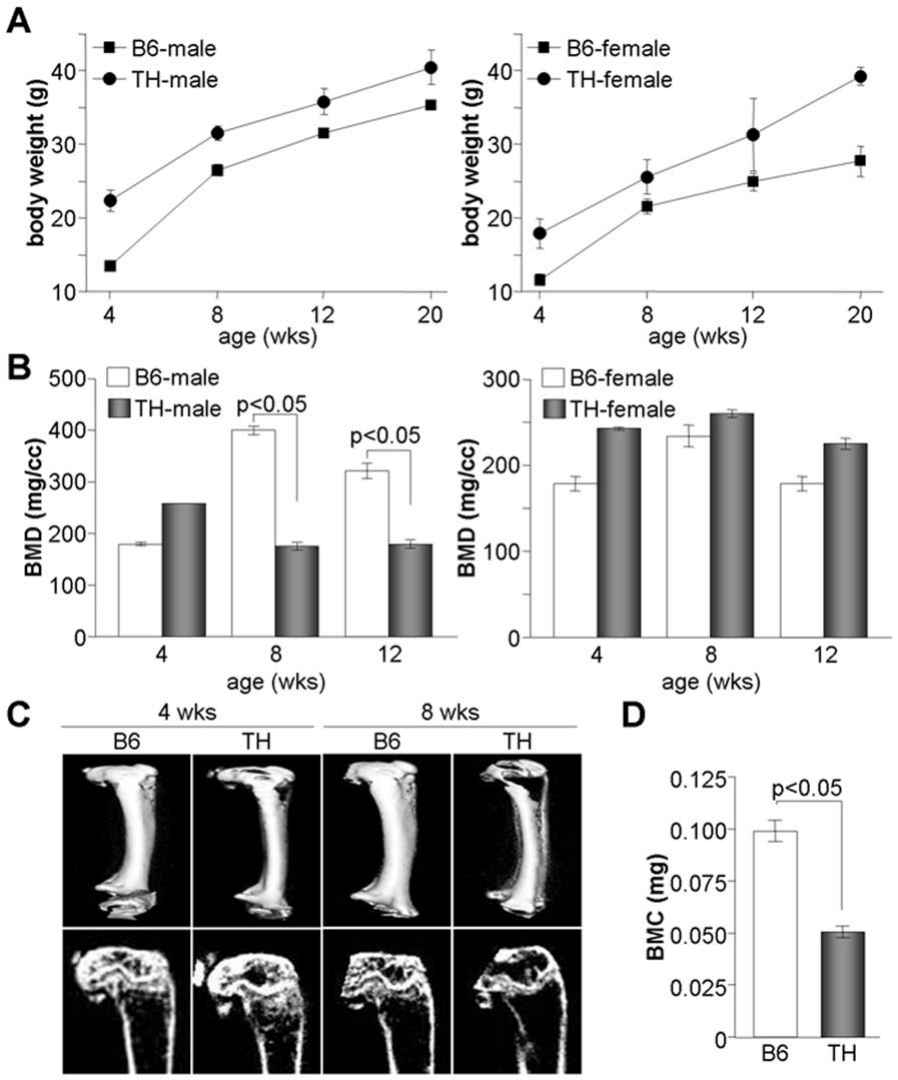
Increased bone loss in male TH mice. (A) Body weights of B6 and TH mice were measured every week from an age of 4 weeks to an age of 20 weeks. The mean values of body weight (n = 6) are presented for both males and females. (B) BMD was determined in males and females at the indicated ages using micro-CT and microView and is expressed as mean ± SEM (n = 7). (C) Three-dimensional micro-CT images of femur and trabecular bone were collected by using a micro-CT scanner and one representative image is shown. (D) Whole femur BMC was analyzed using high-resolution micro-CT, and the result given as the mean ± SEM for seven mice.

Since TH mice are known as a polygenic diabetes and obesity model, we measured gains in body weight in B6 and TH mice fed a normal diet. TH mice experienced significantly increases in body weight compared to age-matched B6 mice during the examination period (Fig. 1A). Although the body weights of male and female TH mice were higher than those of age- and gender-matched B6 mice, BMD was substantially decreased in male TH mice but not in female mice at ages of 8 and 12 weeks (Fig. 1B). Micro-CT scanning also showed a considerable decrease in bone density in TH mice at an age of 8 weeks (Fig. 1C). In addition, bone mineralization was also significantly decreased in male TH mice (Fig. 1D). Previous reports that male TH mice experience increases in blood glucose levels with age and develop hyperglycemia, hyperleptinemia, and hyperinsulemia [31], [32] are consistent with our results and suggest that diabetic phenotypes may be associated with bone loss in male TH mice.

### Osteoblastogenic markers are decreased and osteoclastogenic markers are increased in bone marrow of TH mice

**Figure 2:**
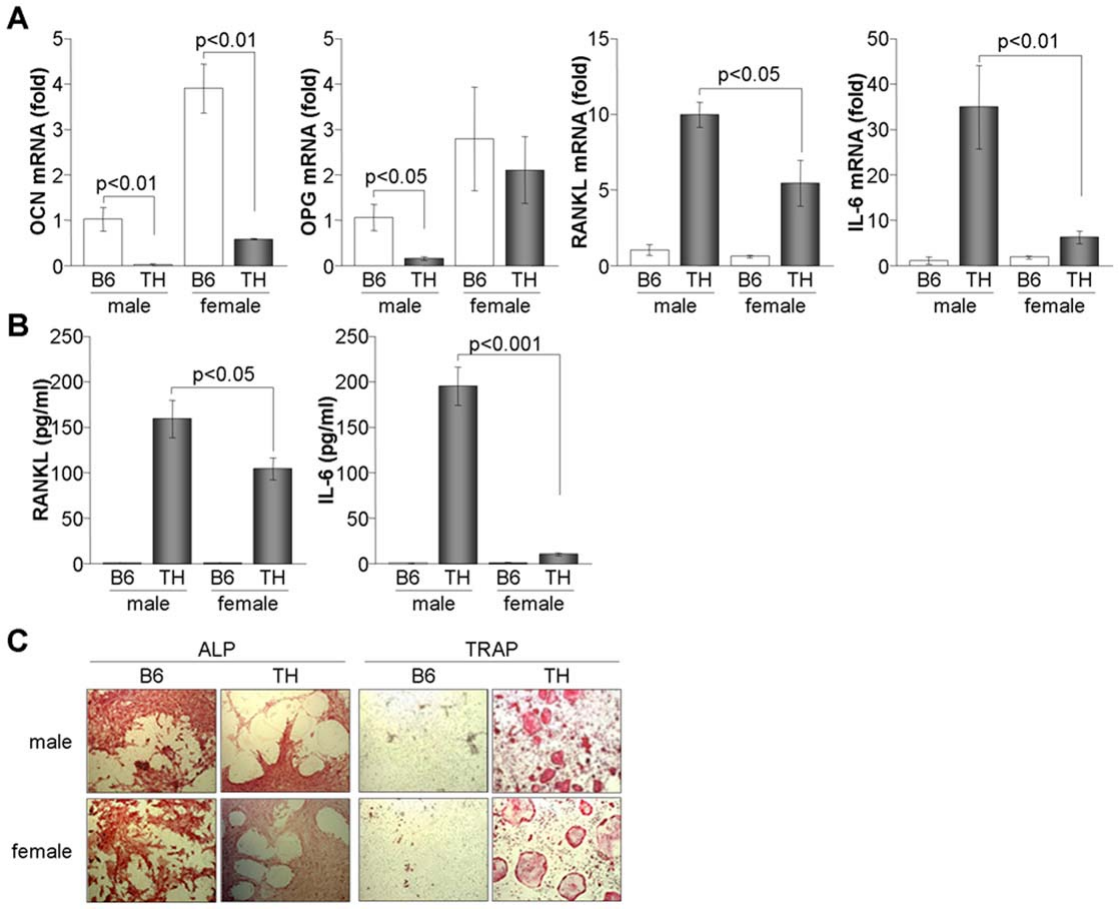
Imbalance of osteoblasts and osteoclasts in bone marrow of TH mice. (A) Total RNA was isolated from bone marrow cells of B6 and TH mice at 8 weeks of age (n = 6) and used for real time-PCR analysis. Relative expression levels of OCN, OPG, RANKL, and IL-6 were calculated after normalization to the level of β-actin. (B) Culture supernatants were collected from bone marrow cells, and RANKL and IL-6 levels were determined by ELISA. (C) Bone marrow cells were isolated and cultured for 24 h. After removal of floating cells, cells were maintained in DMEM supplemented with ascorbic acid (50 µg/ml), β-glycerophosphate (2 mM) and dexamethasone (10 nM) for 8 days. Cells were fixed and stained with either ALP or TRAP.

Attenuation of bone density in TH mice led us to examine the expression levels of osteoblast- and osteoclast-mediated gene markers in bone marrow cells. Osteoblast-specific OCN was significantly decreased in both male and female TH mice compared to B6 mice, whereas OPG was reduced in male TH mice but not in females (Fig. 2A). Moreover, TH mice expressed higher levels of a bone destruction mediating factor, RANKL, and a RANKL inducer, IL-6, in bone marrow. Although female TH mice produced significantly higher levels of RANKL and IL-6 compared to B6 mice, these increases were considerably lower in female than in male TH mice, as evidenced by real time-PCR and ELISA (Fig. 2A and B). Next, we isolated bone marrow cells and cultured the cells under osteoblast differentiation conditions. Osteoblast-expressing ALP was clearly detected in male and female B6 and TH mice, whereas giant multinucleated osteoclasts clarified by TRAP staining were prominently generated in TH mice (Fig. 2C). These results indicate that TH mice expressed increased levels of the osteoclastogenic markers RANKL and IL-6 and reduced amounts of OCN and OPG in bone marrow, resulting in significant bone density loss.

### Hyperleptinemia of TH mice is accompanied by reduction of OCN and substantial increases in IL-6 and IFN-γ

**Figure 3:**
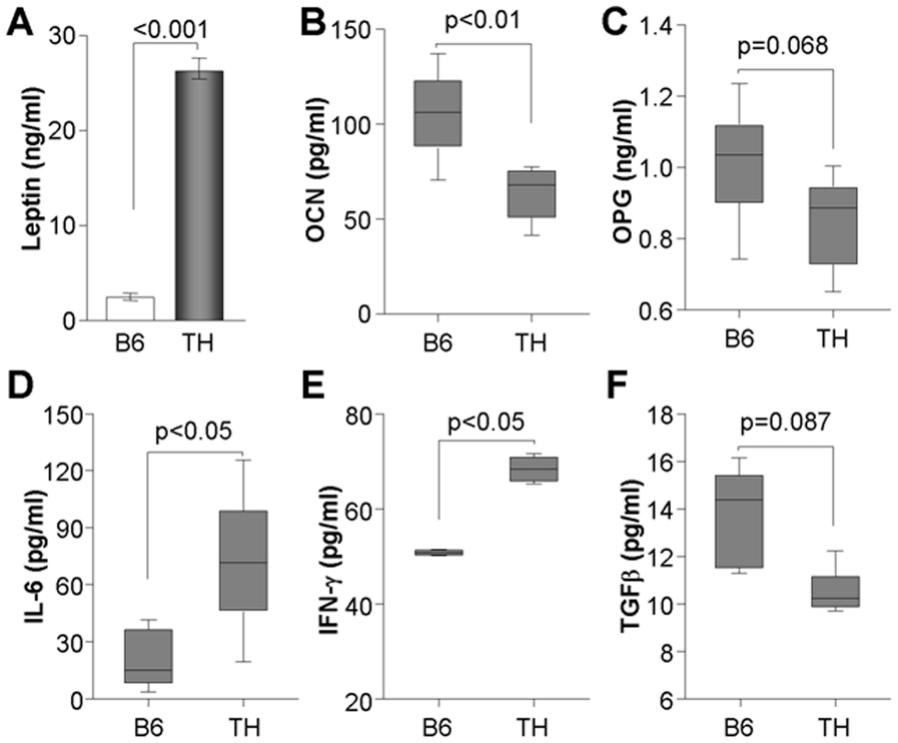
Increased serum levels of leptin and inflammatory cytokines in TH mice. Whole blood was collected from mice (males, n = 6) at 8 weeks and used to measure the serum levels of leptin (A), OCN (B), OPG (C), IL-6 (D), IFN-γ (E), and TGF-β (F). All data are expressed as the mean ± SEM. P value was calculated by student t-test.

Having confirmed the imbalance of osteoblasts and osteoclasts in the bone marrow of TH mice, we further examined the serum levels of osteogenic factors. Using the serum of male B6 and TH mice at an age of 8 weeks, the levels of leptin, osteoblastogenic markers, and osteoclastogenic factors such as cytokines and chemokines were determined by ELISA. TH mice expressed substantially increased amounts of leptin in serum compared to B6 mice, which was consistent with the diabetic phenotype of TH mice (Fig. 3A). Serum OCN expression was also significantly attenuated in TH mice, whereas the reduction of serum OPG was not statistically significant (Fig. 3B and C). Although RANKL expression in TH mice serum was below the threshold of detection of ELISA, serum IL-6 and IFN-γ, both stimulators of RANKL expression, were statistically elevated in TH mice (Fig. 3D and E). Additional analysis revealed that the serum TGF-β level was unchanged in B6 and TH mice (Fig. 3F). Based on the results, we suggest that increased serum levels of the inflammatory cytokines IL-6 and IFN-γ may cause RANKL-induced bone loss in TH mice.

### CD4+ T cells are increased in TH mice and associated with increased IFN-γ and IL-17 production

**Figure 4:**
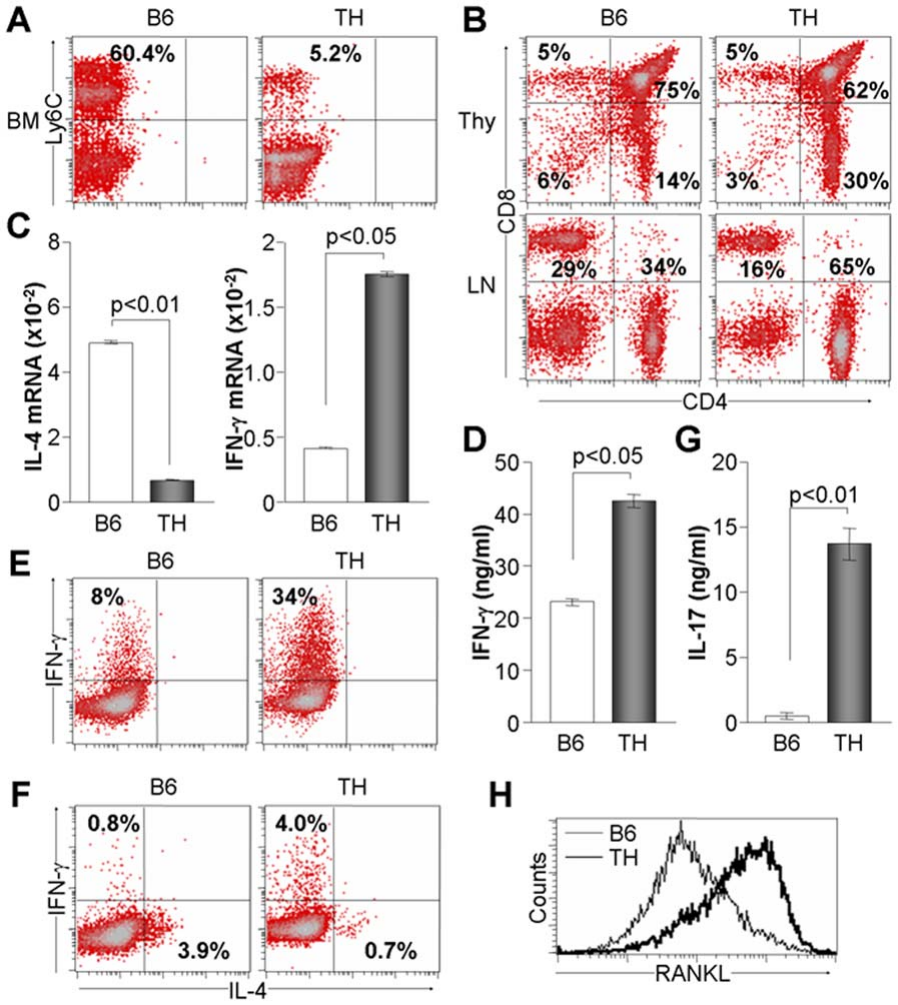
Altered CD4+ T cell functions in TH mice. All B6 and TH mice were analyzed at 8 weeks of age (n = 8). (A) Bone marrow cells were stained with fluorescence-conjugated anti-Ly6C Ab (BD Pharmingen) and analyzed using FACS Calibur and CellQuest program (BD Biosciences). (B) Single cell suspensions of thymus and lymph node were harvested and stained with Abs against CD4 and CD8, followed by flow cytometry analysis. (C) CD4+ T cells were isolated from the lymph node and stimulated with anti-CD3 and anti-CD28 for 24 h. Cells were then collected for real time-PCR to determine the relative expression levels of IL-4 and IFN-γ. (D-F) CD4+ T cells were stimulated for 48 h. IFN-γ production was analyzed in the cell supernatant by ELISA (D) and intracellular cytokine staining (E). (F) T cells were stained with anti-IFN-γ and anti-IL-4 Abs (BD PHarmingen), followed by flow cytometry. (G) T cells were stimulated and subsequently treated with TGF-β and IL-6 for 48 h. IL-17 was measured in the cell supernatant by ELISA. (H) T cells were stimulated with anti-CD3/28 for 48 h, followed by incubation with anti-RANKL Ab and flow cytometry analysis.

Increased levels of serum IL-6 and IFN-γ in TH mice prompted us to examine immune cell development in TH mice. A myeloid marker, Ly6C, was prominently decreased in the bone marrow of TH mice (Fig. 4A), which was consistent with a previous finding of attenuated Ly6C expression in diabetes [33]. Further examination of T cell development revealed that CD4+ T cells were increased in the thymus and lymph node of TH mice when compared to B6 animals (Fig. 4B). In addition, stimulation of isolated CD4+ T cells with anti-CD3 and anti-CD28 Abs for 24 h decreased IL-4 and increased IFN-γ expression in CD4+ T cells of TH mice (Fig. 4C). Specifically, CD4+ T cells of TH mice produced more IFN-γ than those of B6 mice, as demonstrated by the addition of IL-12 with anti-CD3/CD28 Abs (Fig. 4D and E). IL-4-producing CD4+ T cells were significantly attenuated in TH mice in response to exogenous IL-4 addition, whereas IFN-γ-producing cells were elevated (Fig. 4F). Moreover, stimulation of CD4+ T cells with anti-CD3/28 Abs in the presence of TGF-β and IL-6 significantly augmented IL-17 production in TH mice (Fig. 4G). Interestingly, RANKL expression was significantly elevated in activated CD4+ T cells of TH mice compared to WT cells (Fig. 4H). These results indicate that CD4+ T cells of TH mice were biased toward differentiation into IFN-γ- and IL-17-producing cells.

### IL-17-producing CD4+ T cells are increased by leptin and responsible for enhanced RANKL expression in TH mice

**Figure 5:**
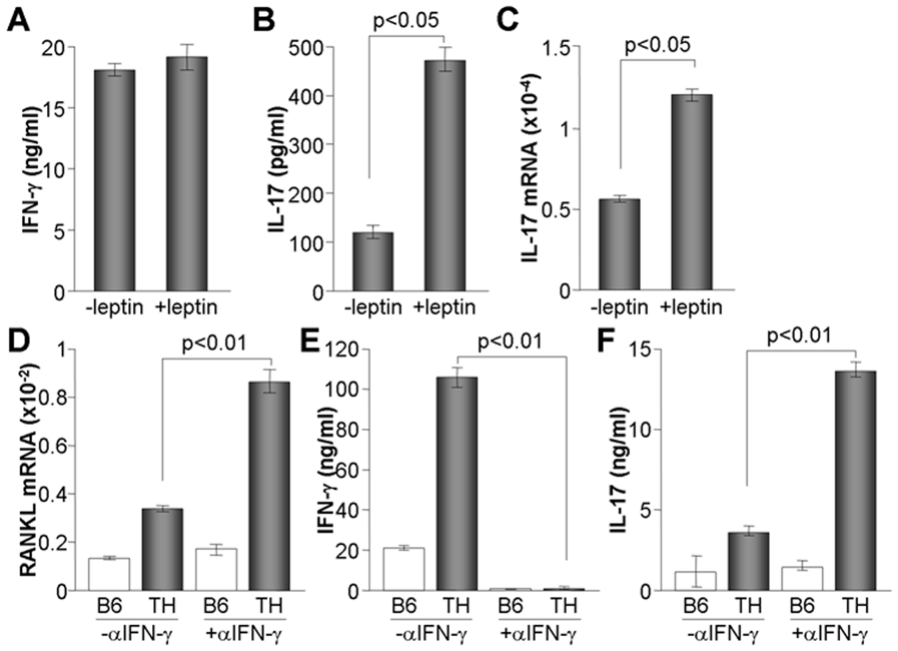
Leptin-mediated IL-17 production in CD4+ T cells. CD4+ T cells were isolated from B6 mice (8 weeks, n = 3) and stimulated with anti-CD3/28 Ab for 24 h. (A-C) Leptin was added to the cells for an additional 24 h. Supernatant was used in ELISA for measurement of IFN-γ (A) and IL-17 (B). Cells were used to determine the relative expression level of IL-17 by real time-PCR (C). (D-F) Stimulated CD4+ T cells were further treated with either control (-αIFN-γ) or anti-IFN-γ Ab (+αIFN-γ) for 24 h and then harvested to determine RANKL expression by real time-PCR (D). Culture supernatants were used to measure the expression of IFN-γ (E) and IL-17 (F) by ELISA.

Since TH mice produced a high amount of leptin and enhanced RANKL expression in CD4+ T cells, we analyzed the direct effects of leptin on the production of IFN-γ and IL-17. Since leptin directly suppresses regulatory T cell proliferation [34], CD4+ T cells were stimulated with anti-CD3/28 Abs for 24 h and then treated with or without leptin for an additional 24 h. Although IFN-γ production was not affected by leptin treatment, IL-17 production was significantly increased (Fig. 5A and B). Leptin directly increased the gene transcription of IL-17 in CD4+ T cells (Fig. 5C). We also assessed whether or not IFN-γ affects RANKL expression in CD4+ T cells. Treatment of CD4+ T cells with anti-IFN-γ Ab increased RANKL expression at the level of gene transcription (Fig. 5D). Blockade of IFN-γ signaling accelerated IL-17 production in CD4+ T cells of TH mice but did not affect IL-17 expression in B6 CD4+ T cells (Fig. 5E and F). We suggest that the increased level of leptin in TH mice preferentially increased IL-17 production and RANKL expression in CD4+ T cells.

### Reduced BMD in TH mice is restored by treatment with alendronate

**Figure 6:**
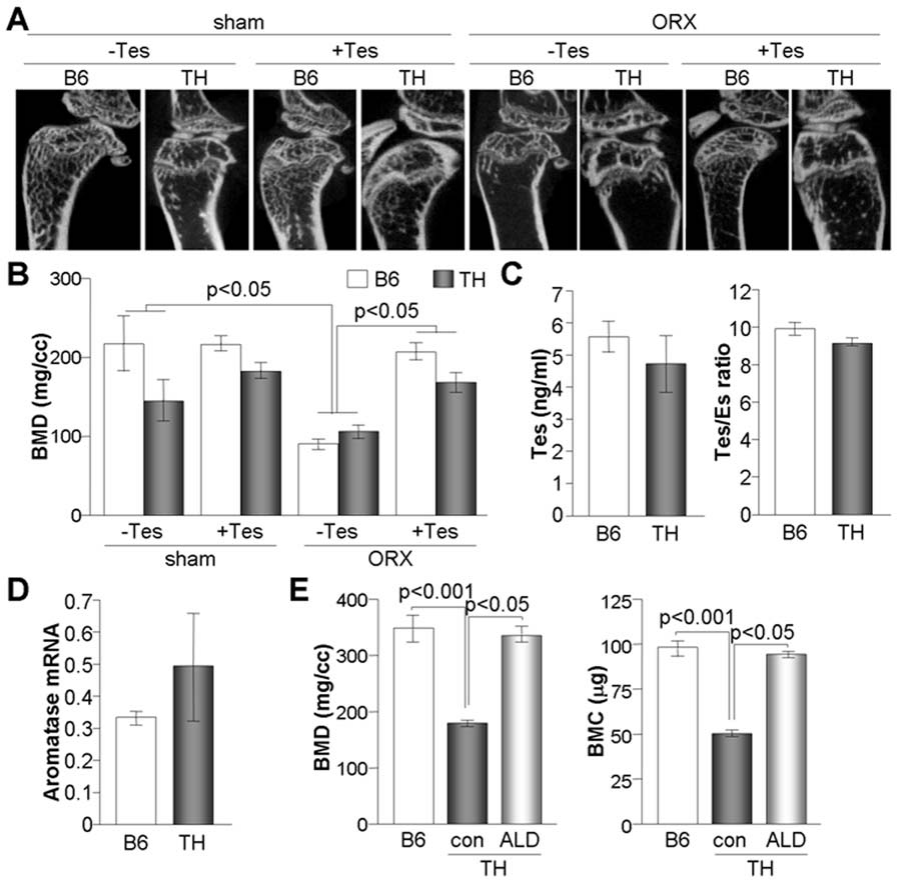
Restoration of BMD in TH mice by testosterone and alendronate. B6 and TH mice (n = 6 each group) were orchiectomized at 8 weeks of age and subsequently injected with vehicle (sesame oil) or testosterone (50 mg/kg) for 6 weeks. (A) Micro-CT images of trabecular bone were reconstructed, and one representative image is presented in each group. (B) BMD was calculated using a micro-CT scanner and micoView. All results are expressed as the mean ± SEM for 6 mice. (C) Serum testosterone levels of 8 week-old WT and TH mice (n =  5) were determined using a high-sensitivity ELISA kit. The testosterone/estradiol ratio was determined after determination of serum estradiol level. (D) Relative expression level of aromatase was quantitated in testis by real time PCR and normalization to the level of β-actin (n = 4). (E) TH mice at 4 weeks of age (n = 7) were orally administered alendronate (ALD, 5 mg/kg/day) for 4 weeks. BMD and BMC were determined in B6 and TH mice at 8 weeks of age using a micro-CT scanner.

Since male TH mice exhibited a considerable bone loss as well as hyperleptinemia, we investigated whether or not the testosterone level changes in TH mice and if this hormone can restore the bone density. First, we injected TH mice with testosterone and examined its effects on bone density. Testosterone did not significantly increase bone density in TH mice, whereas bone loss induced by orchiectomy was restored by the injection of testosterone in both B6 and TH mice (Fig. 6A and B). Furthermore, serum testosterone levels as well as the ratio of testosterone/estrogen were comparable between B6 and TH mice (Fig. 6C). TH mice expressed similar amount of aromatase, which converts testosterone to estradiol and is involved in the control of bone homeostasis (Fig. 6D), suggesting no hormonal defect in osteoporotic TH males. On the other hand, decreased BMD in TH mice was restored by administration with alendronate, which reverses osteoporosis through the activation of osteoblasts and the induction of osteoclast apoptosis (Fig. 6D). These results together suggest that TH mice could be used as a model of secondary osteoporosis to develop novel therapeutics for treating metabolic bone diseases.

## Discussion

In this study, we demonstrated that male TH mice as a polygenic diabetes model developed an osteoporotic phenotype with decreased bone density and increased osteoclastogenic factors, such as IFN-γ, IL-6, and RANKL, in the blood and bone marrow. Moreover, RANKL was prominently increased in CD4+ T cells, which were biased towards differentiation into IL-17-producing Th17 cells in TH mice. Treatment of leptin enhanced IL-17 production *in vitro*.

TH mice have been characterized by hyperleptinemia, hyperinsulemia, hyperlipidemia and male-specific hyperglycemia, and they are established as a polygenic model for type II diabetes with obesity [35]. Insulin deficiency or resistance as well as hyperglycemia in patients with diabetes are known risk factors for metabolic bone diseases, including osteoporosis [29]. Both leptin-deficient ob/ob mice and leptin receptor-deficient db/db mice reveal increased adiposity together with decreased BMD, suggesting an association between adiposity and aberrant BMD [36], [37]. Moreover, T cell inflammation in the adipose tissue of these obese mice reveal increased levels of TNF-α, IFN-γ, or IL-6, resulting in dysregulation of bone homeostasis [38]–[40]. Our results also demonstrate that male TH mice with hyperglycemia and obesity underwent reduction of BMD due to increased T cell inflammation. Although both female and male TH mice are accompanied by obesity and diminished locomotor activity [41], bone loss was specific to male TH mice. Therefore, these results suggest that reduced locomotor activity has no particular effect on BMD diminution in male TH mice. Reduced bone density was the result of decreased levels of OCN and OPG, increased RANKL expression, and high expression of IL-6 in TH mice. Most of all, IL-6 expression was significantly elevated in male TH mice, suggesting an important role for IL-6 in osteoporosis development in male TH mice. IL-6 is critical for the induction of CD4+ T cell differentiation into IL-17-producing T cells [42]. Consistently, TH mice produce a higher amount of IL-17 in activated CD4+ T cells than in WT T cells, and this is well associated with increased RANKL expression in T cells of TH mice. Moreover, leptin directly induces IL-17 production, and blockade of IFN-γ further stimulates IL-17 production and RANKL expression in CD4+ T cells of TH mice [43]. These observations suggest that preferred IL-17 production by CD4+ T cells may increase RANKL-mediated osteoclastic bone destruction in male TH mice.

Osteoporosis is one of the most common chronic bone diseases, involving the progressive loss of bone density with age and sex hormone deficiency. Although osteoporosis is more common in women than men, several risk factors such as alcoholism, inflammatory diseases, and hormonal disorders increase the incidence of secondary osteoporosis in men [44]. In order to prevent and treat osteoporosis, studies on the molecular mechanism of osteoporosis and efficacies of new drugs have been performed using an ovariectomy- or orchiectomy-induced osteoporosis model or a naturally aged SAMP-6 (P6 substrain of senescence-accelerated mice) model [45]. However, these methods are time-consuming and thus extremely costly. Our study shows that TH mice may constitute an effective osteoporosis model as it displays early onset of osteoporosis and male-specific osteoporotic phenotype secondary to hyperglycemia. Moreover, testosterone injection increased BMD in orchiectomized TH mice, and treatment with alendronate restored BMD and BMC values of TH mice to the levels of age-matched B6 mice. Therefore, we propose that TH mice could be a useful animal model of secondary osteoporosis for the development of novel therapeutics that prevent or treat bone loss and bone destruction in metabolic bone diseases.
